# Parents’ preferences and willingness-to-pay for human papilloma virus vaccines in Thailand

**DOI:** 10.1186/s40545-015-0040-8

**Published:** 2015-07-22

**Authors:** Surachat Ngorsuraches, Kornwan Nawanukool, Krittin Petcharamanee, Ungkanit Poopantrakool

**Affiliations:** Department of Pharmacy Administration, Faculty of Pharmaceutical Sciences, Prince of Songkla University, Hatyai, Songkhla 90112 Thailand; Department of Pharmacy Practice, College of Pharmacy, South Dakota State University, Brookings, SD 57007 US

**Keywords:** HPV vaccine, Willingness-to-pay, Patient preference, Discrete choice experiment

## Abstract

**Objective:**

To examine parents’ preferences and willingness-to-pay (WTP) for HPV vaccines.

**Methods:**

A discrete choice experiment (DCE) was used. Parents with at least one daughter aged 9–13 years residing in Songkhla province were asked to choose one alternative from each DCE choice set describing HPV vaccines by four attributes, including cervical cancer risk reduction, genital warts risk reduction, common side effects, and cost. Multinomial logit model was used for data analyses.

**Results:**

Parents preferred higher risk reductions for cervical cancer and genital warts, and lower common side effects. They valued the quadrivalent and bivalent HPV vaccines at 21,189.9 and 10,479.9 Baht, respectively. Results also showed that mothers valued both vaccines more than fathers did.

**Conclusions:**

Parents valued net benefits for both quadrivalent and bivalent HPV vaccines, but they were willing to pay for the quadrivalent vaccine more than for the bivalent vaccine.

## Introduction

Genital human papillomavirus (HPV) is a sexually transmitted infection. Even though most HPV infections are transient and spontaneously eliminated by human immune system, it is a leading cause for the development of cervical cancer with high mortality in women [1–3]. Especially, some HPV genotypes, e.g. HPV 16 and 18, increase the risk of severe cervical neoplasia more than others do [1]. In 2012, cervical cancer was one of the most common cancers in women with an approximate 528,000 new cases and an estimated 266,000 deaths worldwide [4]. In the same year, 8184 women were newly diagnosed and 4513 women died, which accounted for 12.2 % of all women cancer deaths in Thailand [4].

Recently, WHO revealed the core principle of a comprehensive approach for cervical cancer prevention and control [5]. Its recommendation was to provide effective interventions across the life course, based on the natural history of the cancer. HPV vaccine is recommended to be a primary prevention for girls aged nine to 13 years while screening should be a secondary prevention for women aged above 30. In developed countries where screening has been a successful prevention approach, they reported that the mortality of cervical cancer considerably dropped down by 80 % [6]. Currently, Thai Ministry of Public Health recommends a prevention strategy of screening every five years with visual inspection with acetic acid (VIA) for women aged 30 to 45 years, followed by Pap smears every five years for women aged 50 to 60 years. This secondary prevention or screening has been a real challenge in the country. Before 2005, the Ministry estimated that only 25 % of women aged 30 to 65 years got a Pap smear screening in preceding five years [7]. The Ministry of Public Health and National Health Security Office initiated an organized cytology-screening project for women aged 35 to 60 years. Later, even though almost 70 % of targeted women across the country were screened, the follow-up data for those with positive test results were incomplete. Some challenges still remain for the screening as a secondary prevention in the country. For instance, a study showed that the knowledge and awareness of cervical smears were low among some women in Thailand [8]. Various fears, including abnormal result, possible pain, and embarrassment, caused them negative attitudes toward the smears. Thai government decided to put further efforts on the second phase of the organized cytology-screening project but the results of this phase have not been reported yet.

For the primary prevention, two HPV vaccines, including Gardasil® and Cervarix®, have been available in Thailand since 2009. Even though WHO recommends pre-adolescent HPV vaccination and those two existing vaccines are for HPV genotype 16 and 18, which are responsible for 73.8 % of invasive cervical cancers in the country [9], they are not yet included in Expanded Program on Immunization (EPI), which aims to make vaccines available to all children, due to economic efficiency and budget impact reasons. Previously, there were three peer-reviewed publications of economic evaluations comparing vaccines with screenings [10–12]. All studies indicated that the HPV vaccines could be cost-effective under certain vaccine costs. Later, the vaccine costs were declined to the level that made them to be cost-effective. However, their budget impacts were relatively high or their costs were not yet at an affordable level in policy makers’ perspective. Therefore, the screening is still the prevention of choice in Thailand.

Several countries cannot afford HPV vaccines as well, but some of them receive assistance. In 2000, the Global Alliance for Vaccines and Immunization (GAVI) was formally established to help children across the globe to access vaccines. GAVI has aimed to support the vaccinations for 30 million girls in 40 countries by 2020 [13]. After GAVI successfully negotiated vaccine prices with manufacturers, it began to provide vaccines to eight low-income countries including Kenya, Ghana, Lao PDR, Madagascar, Malawi, Niger, Sierra Leone and the United Republic of Tanzania and expected to provide vaccines for 10 more countries in 2014 [13, 14]. It has been a long haul negotiation of HPV vaccines in Thailand and they have been brought to discussions among policy makers several times. The policy makers tried to reduce prices offered by manufacturers, but the negotiation had never come to any conclusion. However, it became a concern that while girls in other countries, including some low-income countries, can access the HPV vaccines, those in Thailand, which is a middle-income country and is not qualified for getting supports from GAVI, cannot. Also, from healthcare providers’ perspective, a study showed that approximately 80 % of nurses and 63 % of doctors supported HPV vaccination [15].

During this long haul, these two HPV vaccines have been available in private insurance and out-of-pocket markets. The manufacturers of these vaccines have made some revenues from those markets, which reflected that some parents were willing to pay for the vaccines. This wiliness-to-pay (WTP) could be an explanation for the long haul. However, it has never been examined rigorously. Also, if the country understands how parents, as taxpayers, view or are willing to pay for the HPV vaccines, policy makers then can consolidate parents’ views into their own views to design better policy. Previously, there was a study that showed 41 % of parents would like their children to be vaccinated [16]. Those parents were directly asked about their WTP for the vaccines, which was subject to some biases. Another similar study recently showed almost 70 % of parents would be willing to pay for the vaccines [17]. The results were subject to some biases as well since the study asked the parents to choose their WTP from arbitrarily specified ranges. Therefore, this study intended to adopt a discrete choice experiment (DCE) to examine parents’ preferences toward the attributes of HPV vaccines and to calculate their WTP. Theoretically, this method is superior since it can be used to estimate WTP more efficiently by using a smaller sample size and it provides greater explanation to allow parents’ more understanding of choices [18]. The DCE also does not require parents to arbitrarily assign their WTP numbers as other methods usually do.

## Methods

In general, a DCE describes various choice sets of a study intervention by its attributes, e.g. efficacy, side effects, and costs. Each choice set contains various hypothetical alternatives with different attributes and levels, randomly combined by a rigorous method of DCE study design. Respondents are asked to choose one alternative that they prefer from each choice set. Finally, statistical analysis based on Random Utility Theory is used to determine the influences of attributes on respondent preference. This study followed a user’s guide published by Lancsar and Louviere [18].

### Attributes and levels

We reviewed the clinical literatures of HPV vaccines to develop a list of attributes for this study [3, 4, 7, 9]. Generally, patients and clinical experts should be interviewed to confirm the validity of this list. However, we replaced these interviews with obtaining attributes from qualitative interview, previous DCE, and economic evaluation types of studies on HPV vaccines since they already extracted information from patients and experts [10–12, 15, 16, 19–22]. Finally, we identified four attributes − cervical cancer risk reduction, genital warts risk reduction, a common side effect, and cost − as not only the best description of HPV vaccines but also important for parents (Table 1). Level ranges were obtained from the same literatures. For the cost attribute, it was stated as parents’ out-of-pocket cost.

**Table 1:** Attributes and levels for HPV vaccine

Attributes	Levels
Cervical cancer risk	0, 2, 4 in 1,000
Genital wart risk	0, 50, 100 in 1,000
Common side effect e.g. mild fever, little pain	2, 6, 10, 14 in 100
Costs for 3 doses of HPV vaccine^a^	0 Baht, 5,000 Baht, 10,000 Baht

^a^The exchange rate was approximately 33 Baht per US$1 [35]

### Discrete choice experiment questionnaire design

**Fig. 1:**
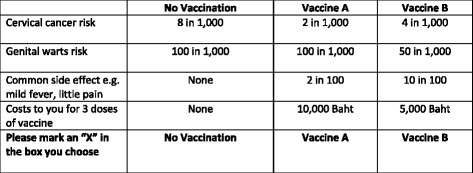
Choice set example

From all possible combinations of selected attributes and levels (3x3x4X3), it was not feasible to present them to an individual respondent. An orthogonal and level balance design was used to randomly draw a subset of all combinations by using Ngene® software (version 1.1.1). In this study, 36 choice sets were generated and divided into six blocks. A self-administered questionnaire was developed in Thai language. Each questionnaire contained six choice sets from each block. Therefore, we had six different questionnaires. Each choice set consisted of two alternatives describing hypothetical vaccines and a reference alternative explaining the effects of no vaccination. Figure 1 shows an example of the choice sets. Another choice set was added to every questionnaire for a validity check. The validity check choice set contained a dominant alternative (highest efficacy, lowest side effect, and no cost), which must be chosen by respondents who understood the questions. All DCE questions were examined for respondents’ understandings by think aloud method with five purposive sampled respondents. Questions on respondents’ characteristics and experiences related to HPV vaccine were included in the questionnaire. Three faculty members at Department of Pharmacy Administration, Faculty of Pharmaceutical Sciences, Prince of Songkla University were asked to check the content validity of the questionnaire before it was piloted with 30 respondents. No major problem was found.

### Data collection

In January 2014, parents with at least one daughter aged 9–13 years residing in Songkhla province were conveniently sampled from public areas where they needed to wait for some purposes, e.g. bus stations, schools, etc. This specific age range was chosen because WHO Guidance note recommended HPV vaccination for girls at these ages [23]. Previously, samples in most studies of parents’ preferences were only mothers or mostly mothers [20, 21, 24]. However, one of these studies indicated that almost all mothers would make vaccination decision with their spouses [21]. To examine real parents’ preferences, this study included both mothers and fathers. Since the choice sets used in this study were unlabeled choices, there was no sample size calculation formula [19]. According to a good research practice, the complete and valid responses of mothers and fathers were aimed at least 150 each to ensure study precision [25]. The ethics committee of Faculty of Pharmaceutical Sciences, Prince of Songkla University approved the study proposal. Verbal consent was asked from every respondent before data collected.

In the questionnaire, respondents received a brief description of HPV infection and its consequences. Descriptions of the choice task, attributes and levels, and an example of a completed choice set were included. Each respondent received only one questionnaire, which contained a total of seven choice sets.

### Data analyses

All data analyses were conducted for father, mother, and overall groups. Based on Random Utility Theory, respondents’ responses for each choice set were observed and analyzed in DCE [18]. The following utility, that a respondent i assigned to an alternative j, U_ij_, was estimated:

$$ {\mathrm{U}}_{\mathrm{ij}} = {\upbeta}_0 + {\upbeta}_1{\mathrm{Cervic}}_{\mathrm{j}} + {\upbeta}_2\ {\mathrm{Warts}}_{\mathrm{j}} + {\upbeta}_{3\ }\mathrm{S}\mathrm{E} + {\upbeta}_4\ {\mathrm{Cost}}_{\mathrm{j}} + {\upvarepsilon}_{\mathrm{ij}} $$

where β_0_ is the constant reflecting respondents’ preference for selecting vaccination relative to no vaccination, β_1_, β_2_, β_3_ are the coefficients or the mean attribute weights of cervical cancer risk (Cervic), genital warts risk (Warts), side effect (SE), and cost (Cost), respectively, ε_ij_ is error term. Multinomial logit model by using Nlogit® version 4 was used to estimate the utility model. The value of each coefficient indicated the relative importance of each attribute, while the sign of the coefficient reflected whether the attribute had a positive or a negative effect on utility or preference, as compared with the base level. The level of statistical significance was set at .05.

Marginal WTPs of the attributes were calculated by taking the ratio of the mean attribute coefficient to the mean coefficient of cost attribute. Each of them represented how much one was willing to pay for a one-unit change in the attribute [18]. Krinsky and Robb method was used to estimate 95 % confidence intervals of WTPs of the attributes [26]. Finally, WTP for both existing HPV vaccines were calculated by multiplying the marginal WTP for that vaccine with the difference between attribute levels of having the vaccine and no vaccination, which were generally obtained from clinical literatures [22, 27].

## Results

### Parents’ characteristics

A total of 400 questionnaires were distributed to parents; 150 and 164 were returned with complete responses, including correct responses on the choice set for validity test, from fathers and mothers, respectively. All 314 responses (78.5 %) were included for data analyses. Table 2 shows parents’ characteristics and experiences related to HPV or HPV vaccine. The overall study respondents’ average age was 42.5 years old and both fathers’ and mothers’ average ages were similar. Less than 30 % were single parent. In average, they were of two children. The majority of respondents had college/university degree or higher and the number of fathers with this degree level seemed to be slightly higher. Most of them either worked for private firms or had their own business and had monthly incomes less than 20,000 Baht. More than a half of mothers had cervical cancer screening before and heard about HPV vaccine. Less than 20 % of overall respondents had relatives, spouses, or friends who had cervical cancer or genital warts before. Interestingly, only almost 70 % of overall respondents’ children had all necessary vaccines. More than a half of mothers stated they themselves decided about vaccination for their children, while approximately 45 % of fathers in this study mentioned they did.

**Table 2:** Respondents’ characteristics and experiences related to HPV or HPV vaccine

	Fathers	Mothers	All
	(*N* = 150)	(*N* = 164)	(*N* = 314)
Age (years, mean ± standard deviation)	43.5 ± 7.5	41.7 ± 7.1	42.5 ± 7.4
Marital status (N, (%))			
Married	114(76.0 %)	123(75.0 %)	237(75.5 %)
Widowed/Divorced/Separated	36(24.0 %)	41(25.0 %)	77(24.5 %)
Number of children (mean ± standard deviation)			
Girls	1.4 ± 0.6	1.5 ± 0.7	1.4 ± 0.7
Total	2.0 ± 0.9	2.2 ± 0.9	2.1 ± 0.9
Educational level (N, (%))			
Primary school	22(14.7 %)	38(23.2 %)	60(19.1 %)
Secondary school	28(18.7 %)	31(18.9 %)	59(18.8 %)
High school/Diploma	38(25.3 %)	38(23.2 %)	76(24.2 %)
College/university or higher	62(41.3 %)	57(34.7 %)	119(37.9 %)
Occupation (N, (%))			
Civil servant	20(13.3 %)	21(12.8 %)	41(13.1 %)
Private firm	54(36.0 %)	52(31.7 %)	106(33.8 %)
Own business	51(34.0 %)	55(33.5 %)	106(33.8 %)
Others	25(16.7 %)	36(21.9 %)	61(19.4 %)
Monthly household income (N, (%))			
Up to 10,000 Baht	52(34.7 %)	58(35.4 %)	110(35.0 %)
10,001 to 20,000 Baht	38(25.3 %)	46(28.0 %)	84(26.7 %)
20,001 to 30,000 Baht	35(23.3 %)	33(20.1 %)	68(21.7 %)
30,001 to 40,000 Baht	16(10.7 %)	18(10.9 %)	34(10.8 %)
Higher than 40,000	9(6.0 %)	9(5.5 %)	18(5.7 %)
Experience in cervical cancer screening (N, (%))	-	90(54.9 %)	90(54.9 %)
Family members, spouses or friends had cervical cancer/genital warts (N, (%))	21(14 %)	39(23.8 %)	60(19.1 %)
Heard about HPV vaccine (N, (%))	66(44.0 %)	84(51.2 %)	150(47.8 %)
Your child received other necessary vaccines.			
All	93(62 %)	122(74.4 %)	215(68.5 %)
Some	44(29.3 %)	33(20.1 %)	77(24.5 %)
Don’t know	13(8.7 %)	9(5.5 %)	22(7.0 %)
Person who decided about vaccination for your child			
You	68(45.3 %)	84(51.2 %)	152(48.4 %)
Your spouse	17(11.3 %)	15(9.2 %)	32(10.2 %)
You and your spouse	63(42 %)	65(39.6 %)	128(40.8 %)
Others	2(1.3 %)	-	2(0.6 %)

The results of the multinomial logit model are presented in Table 3. All parents seemed to understand the choice tasks well since there was no bad observation, including always chose one alternative. Only 7 % of all observations chose no vaccine alternative, while approximately 49 % and 44 % chose the first and second alternatives in the choice sets.

**Table 3:** Results from the multinomial logit model

Attributes	Fathers^a^		Mothers^b^		Overall^c^	
	Estimates	Standard error	Estimates	Standard error	Estimates	Standard error
Constant	0.48206	0.26734	0.89736	0.26769	0.70222	0.18843
Cervical cancer risk	0.33480	0.03580	0.26825	0.03364	0.29818	0.02436
Genital warts risk	0.01145	0.00137	0.01536	0.00134	0.01330	0.00095
Common side effect	−0.08724	0.01287	−0.08055	0.01268	−0.08346	0.00903
Cost	−0.00011	0.00001	−0.00012	0.00001	−0.00011	0.00001

^a^Log-likelihood = −684.57, Akaike information criterion = 1.82, Pseudo-R^2^ = 0.16

^b^Log-likelihood = −706.19, Akaike information criterion = 1.45, Pseudo-R^2^ = 0.18

^c^Log-likelihood = −1,400.42, Akaike information criterion = 1.49, Pseudo-R^2^ = 0.16

*P* < 0.0001 for all estimates, except constant estimate for fathers *p* = 0.07

All estimated coefficients had expected signs and were statistically significant in every model. The positive constant in the parent’s model implied that they generally preferred getting their children vaccinations, although fathers’ model showed the constant was significant only at only *p* < 0.1. The positive signs of the cervical cancer and genital warts risk parameters indicated that the parents preferred vaccines with higher reduction of these risks. The negative signs of the common side effects and cost parameters reflected that they preferred lower side effects and paying less money for vaccines.

The coefficient strength of each attribute can be interpreted as its preference weight on parents’ utility when other attributes are assumed constant. According to the coefficient strengths on the same scales of the cervical cancer risk and genital warts risk, parents weighted on reducing cervical cancer risk more than reducing genital warts risk, as indicated by the higher coefficient value (0.298 VS 0.013). The results from fathers’ and mothers’ models were relatively similar with a note that fathers put slightly higher weight on the cervical cancer risk than mothers did.

Table 4 shows the WTP for individual attributes of HPV vaccine. The parents’ preferences for both vaccine effectiveness and common side effects were put in WTP space to make easier for comparison. Marginal WTP for each attribute was calculated to reflect how much respondents were willing to pay for a unit change of each attribute. The results showed that parents were willing to pay for 6240.4 Baht for their child’s HPV vaccination when everything else was equal, as compared to no vaccination. Interestingly, compared to fathers, mothers were willing to pay 3546.7 Baht more for their daughters’ vaccinations. Parents were willing to pay 2671.5 and 119.0 Baht for a one in 1000 person reduction in the number of persons having cervical cancer and genital warts, respectively. On the other hand, they were willing to pay 746.7 Baht for one person in 100 persons to avoid common side effects from getting vaccinated. Interestingly, while both fathers and mothers were willing to pay approximately same amount for avoiding one unit change of genital warts risk and common side effects, fathers intended to pay 416.5 Baht more than mothers did for avoiding one unit change of cervical cancer risk.

**Table 4:** Willingness-to-pay for the attributes of HPV vaccine

Attributes	Average Willingness-to-pay (95 % confidence interval) (Baht)		
	Fathers	Mothers	All
Constant (reference: no vaccination)	4,284.7 (−427.5 to 8,935.1)	7,831.4 (3,312.4 to 12,533.54)	6,240.4 (3,013.6 to 9,501.3)
Cervical cancer (1 in 1,000 risk reduction)	3,038.0 (2,234.7 to 4,076.0)	2,621.5 (2,366.3 to 3,207.0)	2,671.5 (2,142.0 to 3,297.8)
Genital warts (1 in 1,000 risk reduction)	103.6 (73.9 to 141.4)	135.2 (102.2 to 179.0)	119.0 (96.2 to 146.5)
Common side effect (1 in 100)	−789.8 (−1,098.6 to −538.5)	−708.9 (−987.6 to −478.4)	−746.7 (− 949.0 to −571.7)

Table 5 shows the WTP for quadrivalent and bivalent HPV vaccines. Attribute levels of both types of vaccines were obtained from Oteng et al. and Centers for Disease Control and Prevention (CDC) to calculate the WTP for both vaccines [22, 27]. For parents, results showed that if 55 persons with either quadrivalent or bivalent HPV vaccines per 1000 persons avoided cervical cancer, the value would be 14,693.3 Baht. Also, the value parents would pay 10,453.8 Baht for avoiding common side effects, which occurred approximately 14 % from vaccination. However, the quadrivalent HPV vaccine helped 90 persons per 1000 persons to avoid genital warts as well and the value would be 10,710.0 Baht that parents were willing to pay. Totally, parents valued the quadrivalent and bivalent HPV vaccines at 21,189.9 and 10,479.9 Baht, respectively. The results also showed that mothers were willing to pay 5232.6 and 2388.6 Baht more than fathers were for the quadrivalent and bivalent HPV vaccines, respectively.

**Table 5:** Willingness-to-pay for quadrivalent and bivalent vaccines (Baht)

Attributes	Average WTP for quadrivalent vaccine (95 % confidence interval)			Average WTP for bivalent vaccine (95 % confidence interval)		
	(Baht)			(Baht)		
	Father WTP	Mother WTP	Overall WTP	Father WTP	Mother WTP	Overall WTP
Constant (reference: no vaccination)	4,284.7 (−427.5 to 8,935.1)	7,831.4 (3,312.4 to 12,533.5)	6,240.4 (3,013.6 to 9,501.3)	4,284.7 (−427.5 to 8,935.1)	7,831.4 (3,312.4 to 12,533.5)	6,240.4 (3,013.6 to 9,501.3)
Cervical cancer risk	16,709.0 (12,290.9 to 22,418.0)	14,418.3 (13,014.7 to 17,638.5)	14,693.3 (11,781.0 to 18,137.9)	16,709.0 (12,290.9 to 22,418.0)	14,418.3 (13,014.7 to 17,638.5)	14,693.3 (11,781.0 to 18,137.9)
Genital wart risk	9,324.0 (6,651.0 to 12,726.0)	12,168.0 (9,198.0 to 16,110.0)	10,710.0 (8,658.0 to 13,185.0)	0	0	0
Common side effect	−11,057.2 (−15,380.4 to −7,539.0)	−9,924.6 (−13,826.4 to −6,697.6)	−10,453.8 (−13,286.0 to −8,003.8)	−11,057.2 (−15,380.4 to −7,539.0)	−9,924.6 (−13,826.4 to −6,697.6)	−10,453.8 (−13,286.0 to −8,003.8)
Total WTP	19,260.5(7,846.2 to 31,889.7)	24,493.1 (16,217.7 to 34,882.3)	21,189.9 (13,393.4 to 29,559.5)	9,936.5 (1,195.2to 19,163.7)	12,325.1 (7,019.7 to 18,772.3)	10,479.9 (4,735.4 to 16,374.5)

## Discussions

This study suggested that parents with daughters aged 9–13 years had different preferences for vaccines’ attributes and were willing to trade among these attributes when they chose vaccines for their daughters. The results were intuitive that parents preferred higher vaccine efficacy, lower side effect, and lower cost. Parents highly weighted on more serious efficacy, which was cervical cancer risk in this case, than less serious efficacy, which was genital warts risk. Results showed that fathers put lower weight on vaccination, as compared to no vaccination, and slightly higher weight on the cervical cancer risk, as compared to mothers did. One of the reasons could be that the proportion of fathers in this study, who knew someone who had either cervical cancer or genital warts and knew about HPV vaccine, was less than the proportion of mothers; therefore they might not value the vaccination as much as mothers did. The study result about the knowledge of fathers was consistent with the results from various studies indicating that fathers might have poor knowledge or awareness of HPV vaccine [27–31]. Also, the cervical cancer might seem to be more serious than other attributes to them. Without much knowledge and experience, the fathers’ preferences and WTP for the vaccines could be ambiguous, but it was worth examining here because almost half of fathers and mothers in this study, if not together with their spouses, stated that they made decision regarding vaccination for children in their families.

The parents’ average WTP for the quadrivalent HPV vaccine was extremely high, as compared to its current price (less than 10,000 Baht for three doses) in Thailand. It was much higher than parents’ WTP for the HPV vaccine in two previous studies (about 500–3000 Baht for three doses) [16, 17]. The parents’ WTP for the vaccine among these three studies also did not align with the parents’ monthly household incomes, which should be a major influencer of WTP. One of the reasons could be that all of them used different methods for determining WTP for the vaccine. However, this study was only study that examined WTP by using a rigorous study design, DCE.

Compared to other vaccines and even the prices of other new and expensive vaccines, e.g. invasive pneumococcal disease vaccine (about 12,000 Baht), the parents’ WTP for the quadrivalent HPV vaccine was still higher. Their WTP for the bivalent HPV vaccine was also relatively high, but closer to the vaccine’s current price in the country. This might be partially accounted for by the fact that cervical cancer in general is a dreaded and fatal disease [25]. Also, previous studies showed most parents were aware of cervical cancer and wanted to vaccinate their daughters [16, 27–31]. In addition, since the wide range of cervical cancer screening coverage (30 % to 75 %) [32] reflected that the access to and/or utilization of the screening were still limited, it could be a reason that the parents in this study were willing to pay relatively high for HPV vaccines.

Compared to Vietnamese mothers’ WTP (about 3200 to 11,000 Baht) for the vaccine concerning only cervical cancer risk reduction and efficacy period [21], which was probably comparable to the bivalent vaccine in this study, the results showed that Thai parents were willing to pay approximately the same amount. This study revealed that the average WTP for the quadrivalent HPV vaccine was approximately 3 % of 2013 annual household income [33], which was slightly lower than the results from the study in Vietnam [21]. Interestingly, the results from the study in Vietnam and this study showed that the average WTP for the bivalent vaccine was very close to its price in own country.

In Thailand, there has been a substantial effort and debate to include the HPV vaccines in the EPI. Even though currently the vaccine prices are approximately the cost-effective prices from the economic evaluation studies, they still have large impact on health care budget. Also, all screenings are still effective alternatives for cervical cancer prevention. In 2012, when the Ministry of Public Health proposed to pay for 500 Baht per dose, HITAP suggested that the cost-effective price of HPV vaccine should not exceed 190 Baht per dose [34]. Certainly, the average WTPs for HPV vaccines in this study would appear to be much inflated in the eyes of policy makers in the country. However, the results were similar to the results in a US study, which showed that the WTP for the quadrivalent HPV vaccine was almost double its current retail price [20].

Ones must “not” interpret the WTP calculated in this study as an appropriate or reasonable market price. Instead, using the same explanation as discussed in a previous study [20], as a simple cost-benefit analysis, the interpretation of the results should be that Thai parents valued the benefits of quadrivalent HPV vaccine more than its cost or they realized net benefits of the vaccine, which meant the vaccine were worth their value of money. This interpretation was consistent with the previous economic evaluation studies indicating that the vaccine could be cost-effective under certain vaccine prices [10–12]. Similarly, the parents in this study were willing to pay for the bivalent HPV vaccine approximately at the vaccine price or slightly higher. It is worth mentioning here that these parents had relatively not high household incomes and they still were willing to pay at these prices. Therefore, it was possible that manufacturers could already recognize parents’ WTP for the vaccines and attempt to maximize their profits. They might adopt the concept of WTP in price discrimination and enjoyed their sales in private or out-of-pocket markets. When policy makers tried to negotiate for low price, the manufacturers never agreed with that price and did not put on effort as much as they usually did for other drugs or treatments. Therefore, as long as policy makers are confident with the effectiveness of the screenings, they should try harder to educate the public regarding the screening for cervical cancer because it would not only reduce the risk of cervical cancer but also increase their power of price negotiation because of a decrease in parents’ WTP for the HPV vaccines in private or out-of-pocket markets.

This study suffered from some limitations. First, even though DCE used in this study is a state-of-the-art stated preference method, ones may always argue that it does not reveal or reflect true preference or value since all decisions are not really made. However, this study already tried to minimize validity threats as many as possible, e.g. providing an opt-out alternative in each choice set. Second, all choice sets comprised only limited number of vaccine attributes from literatures. There were other attributes that could also affect parents’ preferences. Further research should adopt qualitative interviews to focus on parents’ views on the vaccine attributes. Third, the inclusion of the low risk reduction of cervical cancer might be too difficult for parents to understand its real meaning. Finally, this study examined preferences among parents in a Southern province in Thailand, the results could not be directly generalized to parents in the whole country since they might have different socioeconomic characteristics, especially income and education. For instance, the country’s average household income was approximately 25,000 Baht [33], which was higher than the majority of parents’ household income in this study. On the other hand, this study had higher percentage of parents with college/university or higher degree, which could also induce response bias. Consequently, these characteristics would affect their preferences and WTP.

## Conclusions

This study revealed that parents weighted on the risk reduction of cervical cancer and common side effects more than they did on the risk reduction of genital warts when they chose their preferred HPV vaccine. They valued net benefits for both quadrivalent and bivalent vaccines, but they were willing to pay for the quadrivalent vaccine more than for the bivalent HPV vaccine due to the additional benefit against genital warts.
